# Predictors of cross-lingual competence in Australian mental health professionals

**DOI:** 10.1080/00049530.2026.2640243

**Published:** 2026-03-11

**Authors:** Marta Garcia de Blakely, Jaimee Stuart, Nicola Sheeran

**Affiliations:** a School of Applied Psychology, Griffith University, Brisbane, Australia; b United Nations University Institute in Macau, Macau SAR, China; c School of Psychology and Public Health, La Trobe University, Bundoora, Victoria, Australia

**Keywords:** Cross-lingual competence, cultural competence, mental health practitioners, psychologists

## Abstract

**Objective:**

Although spoken language plays a crucial role in psychotherapy with clients whose native language differs from the language of therapy, research on mental health practitioners’ language proficiency remains scarce. Our study explored predictors of MHP’s self-reported competence in cross-lingual practice.

**Method:**

Four hundred and fourteen MHP’s working in Australia, completed an online survey measuring key features of cross-lingual competence (self-perceptions, knowledge of client difficulties, barriers imposed by language), as well as engagement in multicultural training, experience with culturally and linguistically diverse (CALD) clients, and demographic characteristics.

**Results:**

Regression analyses indicated that engagement with CALD clients, being bi-lingual, having had multicultural training, and being fully registered significantly predicted self-perceived competence. However, only engagement with CALD clients predicted perceptions of difficulties faced by clients, and being monolingual and greater engagement with CALD clients predicted perceptions of barriers for MHP’s.

**Conclusions:**

Engagement with CALD clients was associated with greater cross-lingual competence regardless of the ethnic status of the clinician. However, specific training in how to work in a cross-lingual context is required to ensure that all clinicians have strategies to manage the barriers they and their client’s may face.

## KEY POINTS

**What is already known about this topic:**

1. Most mental health professionals (MHPs) in Australia speak English and provide services in English to clients who have English as a second language.
2. Most multicultural training does not explicitly teach skills related to working cross lingually.
3. Little is known about how competent Australian MHPs are to provide cross lingual psychotherapy nor what predicts cross-lingual competence.

**What this topic adds:**

1. Engagement in multicultural training was associated with MHP’s perceived cross-lingual competence but not their knowledge of difficulties/barriers client’s and professionals may face.
2. Greater exposure to CALD clients was associated with both MHP’s perceived cross-lingual competence and knowledge-based competence in terms of difficulties/barriers client’s and professionals face in cross-lingual therapy.
3. Clinician’s ethnic status did not predict self-perceived competence, but monolingual’s who worked more frequently with CALD clients perceived greater barriers to cross-lingual work.

Australia is one of the most multicultural and multilingual societies in the world, with almost half (49%) of its population either born overseas or having at least one parent born abroad; many from non-English speaking countries (Australian Bureau of Statistics, 2021). As a result, mental health professionals are increasingly attending to the psychological needs of people from culturally and linguistically diverse backgrounds across a range of settings (i.e., schools, community centres, clinics, hospitals) (Milner & Khawaja, 2010). It is increasingly vital to ensure that therapeutic environments acknowledge and integrate diverse cultural perspectives and lived experiences, as reflected in polices, guidelines, and accreditation standards at federal (e.g., The National Standards for Mental Health Services; Commonwealth Government of Australia, 2010; Professional competencies for psychologists; Psychology Board of Australia, 2025a), organisational (e.g., Australian Psychological Society), and individual practitioner levels (Lee & Khawaja, 2013; Minas et al., 2013). This includes culturally and linguistically diverse (CALD) individuals from migrant and refugee backgrounds, as well as international students and temporary residents in Australia (Psychology Board of Australia, 2025a).

Despite English being Australia’s official language, more than 300 languages are spoken across the country. Census data indicate that only 40%–50% of migrants report speaking English very well, with 2%–3% not speaking English at all (Australian Bureau of Statistics, 2021). Yet, English is the primary language in many therapeutic settings. When clients and therapists do not share the same native language, and psychological assessment and psychotherapy occur in the client’s non-native language, the dynamic is referred to as *cross-lingual psychotherapy* (Stevens & Holland, 2008). This can be challenging for both clients and practitioners, and language barriers can put clients at a disadvantage as there are increased challenges in communicating the nuance of symptoms and complex needs.

In this paper, *language diversity* specifically refers to the native languages, other than English, spoken by culturally diverse clients. Existing, albeit limited, research consistently identifies language diversity as a barrier to accessing and utilising available mental health services (Garcia de Blakeley et al., 2017; Khawaja & Stein, 2016; Menon et al., 2025; Wohler & Dantas, 2017). Crucially, the under-utilisation of mental health services by culturally and linguistically diverse (CALD) individuals is linked to a lack of linguistically competent services and insufficient skills among mental health professionals (Minas et al., 2013).

Language diversity can significantly impact on the effectiveness of clinical processes, regardless of a client’s language proficiency. When therapy is conducted in a client’s non-native language, several aspects can be affected including the way emotions are experienced and expressed (Kheirzadeh & Hajiabed, 2016; Verkerk et al., 2023) and the ability to recount and retrieve traumatic, autobiographical, and general memories (Schroeder & Marian, 2014; Schwanberg, 2010). Moreover, the therapeutic alliance may be weakened (Kokaliari et al., 2013; Stevens & Holland, 2008) and assessments can be affected, potentially leading to inaccurate diagnoses (Erkoreka et al., 2020).

To manage these challenges, it is typically recommended that linguistically diverse clients are either matched with a therapist who speaks their language or use interpreters. However, these options have their own challenges (Tan & Denson, 2019), are not always feasible, and clients who are judged “fluent enough” are typically provided psychological assessment and/or treatment in English, their non-native language (Rolland et al., 2021). Given cross-lingual psychotherapy is the most frequently used model to deliver psychological services to linguistically diverse clients it is important to ascertain how competent MHPs are to provide cross-lingual services to their linguistically diverse clients.

Despite language being fundamental to Western psychotherapies and particularly crucial in cross-lingual work, there has been surprisingly little attention given to guidelines, research, or practice on developing and assessing mental health professionals’ competence in this area. When language competence is mentioned, it is typically only a brief inclusion within the broader topic of multicultural competence.

Multicultural competence has been conceptualised using a tripartite framework emphasising the qualities of the MHP in terms of (1) their cultural awareness (attitudes and beliefs), (2) their cultural knowledge, and (3) their cultural skills (Sue, 2001). However, there are ongoing concerns about a lack of definitional clarity and agreement on what constitutes multicultural awareness, knowledge, skills, and multicultural competence in general, resulting in debates on how to reliably and validly measure competence (see Sue et al., 2009). Current measures of multicultural competence assess attributes known to be present and effective when working with CALD clients and are typically self-report (i.e., Multicultural Counselling Inventory (MCI) Sodowsky et al., 1994, pp.; Multicultural Mental Health Awareness Scale Khawaja et al., 2009) or else observer, supervisor or client rated (i.e., the Cross-cultural Counselling Inventory-Revised (CCCI-R) LaFromboise et al., 1991). Factor structures vary from a single factor thought to represent “general multicultural competence” (e.g., CCCI-R) to multi-factor representing “knowledge, awareness, skills, and therapeutic alliance” (e.g., MCI), demonstrating the multifaceted nature of the construct.

Studies exploring MHP’s competence in working with multicultural clients have identified a number of characteristics that predict self-reported competence including their ethnic status (e.g., self-identification as belonging to an ethnic minority) (Dune et al., 2022; Fuertes & Brobst, 2002; Gonzales et al., 2022; Hill et al., 2013; Sodowsky et al., 1998), frequency of work with culturally diverse clients (Arthur & Januszkowski, 2001), engagement in multicultural training (Smith et al., 2006), and engagement in culture focused multicultural supervision (Ancis & Marshall, 2010; Falender et al., 2013). These factors are malleable to change and could guide the development of recommendations for improving MHP’s cross-lingual competence. Similarly, the MHPs’ ethnic and language status (i.e., bilingual), while potentially more stable and less malleable, can increase our understanding of how culturally and/or linguistically diverse practitioners can be best utilised to serve diverse populations. It is currently often assumed that culturally and/or linguistically diverse practitioners are inherently competent to work with clients from diverse cultures simply because they are diverse themselves (Geerlings et al., 2018; Tan & Denson, 2019). Yet, CALD mental health practitioners have reported their own difficulties, and some research finds they report a lack of multicultural competence working with clients from diverse cultures (Drolet et al., 2014; Tan & Denson, 2019). Thus, further understanding of the influence of MHPs minority status on their cross-lingual competence is necessary.

As briefly mentioned, studies on multicultural competence consistently show that multicultural training increases competence (Chu et al., 2022). For example, a meta-analysis of 45 studies demonstrated a moderate effect of training on multicultural competence (Smith et al., 2006) and both self-report and objective measures of competence yielded similar effect sizes. Interventions can range from single workshops to semester long university courses (Castillo et al., 2007; Smith et al., 2006), with several studies demonstrating an effect of “unspecified amounts of ‘previous multicultural training’ (Constantine, 2001; Sodowsky et al., 1998) suggesting that regardless of varied content and duration, training significantly improves MHPs” multicultural competence.

Arthur and Januszkowski (2001) found that alongside multicultural training, frequency of work with CALD clients significantly predicted self-reported multicultural competence. Mental health practitioners who worked with CALD clients more often perceived themselves as more culturally competent than MHPs who worked with CALD clients infrequently. It is well established that positive intergroup contact reduces threat-related anxiety, conflict, and prejudice, while fostering tolerance, empathy, and perspective-taking (Pettigrew & Tropp, 2008). Thus, exposure to diversity itself, beyond that experienced in clinical practice, may contribute to MHPs feeling more familiar and confident, and therefore, more competent in working with CALD clients. However, most research in this area relies on self-report measures of competence, and the actual impact on demonstrated knowledge and skill application remains largely underexplored and unknown.

International studies indicate that mental health practitioners from ethnic minority backgrounds self-report significantly higher multicultural competence than those from majority cultures (Constantine, 2001; Hill et al., 2013; Sodowsky et al., 1998). However, Arthur and Januszkowski (2001) found that although ethnic minority counsellors in Canada reported greater competence, ethnicity ceased to be a significant predictor when considered alongside other factors such as training and CALD caseload.

Australian research on predictors of multicultural competence is limited, typically involving small, exploratory studies with postgraduate students. Findings align with international evidence that ethnic minority status, clinical experience with CALD clients, culture-specific supervision, and training enhance competence (Geerlings et al., 2018; Gonzales et al., 2022; Ho & O’Donovan, 2018; Khawaja et al., 2009; Lee & Khawaja, 2013; Tan & Denson, 2019). Dune et al. (2022) found non-white mental-health professionals were more culturally aware and developed better multicultural counselling relationships. Gonzales et al. (2022) similarly found that multicultural and bicultural MHP’s demonstrated greater multicultural awareness, as did bi- and multilingual MHP’s. Lee and Khawaja (2013) found that internships with CALD clients and culture-specific supervision improved trainees’ self-reported competence, whereas brief lecture-based training did not. Together quantitative and qualitative studies suggest that cross-lingual competence may be strengthened through engagement with CALD clients, language diversity training, and practitioners’ own bilingual status (Bowker & Richards, 2004; Costa & Dewaele, 2014; Gonzales et al., 2022; Ivers & Villalba, 2015).

## The current study

As highlighted above, to the authors’ knowledge no research has considered predictors of cross-lingual competence in MHP’s outside of general multicultural competence. However, research investigating predictors of cultural competence suggests a range of individual characteristics that are mostly malleable and may predict MHP’s self-rated cross-lingual competence. Thus, the aim of this study was to examine whether multicultural training, engagement with CALD clients, professional status, ethnic status, and language status was associated with MHPs’ cross-lingual competence as measured by the perceptions of cross-lingual practice (PCLP) scale. The PCLP was developed in line with Sue (2001) conceptualisation of multicultural competence and comprises three subscales; self-perceived cross-lingual competence, perceived knowledge about difficulties faced by linguistically diverse clients, and barriers faced by MHPs in cross-lingual practice (Garcia de Blakeley et al., 2023).

It was hypothesised that MHPs who belong to an ethnic minority, are bilingual, work with CALD clients more frequently, have had multicultural training, and are fully registered will score significantly higher than their counterparts on the perceptions of competence subscale of the PCLP (H1). It was also hypothesised that these factors would significantly predict self-perceived cross-lingual competence (H2). Less research has considered predictors of MHP’s perceived knowledge about difficulties faced by clients who are linguistically diverse or knowledge of the barriers they may face themselves. As such two research questions were proposed:

RQ1: To what extent does multicultural training, engagement with CALD clients, professional status, ethnic status, and language status predict MHP’s perceptions of the difficulties faced by clients.

RQ2: To what extent does multicultural training, engagement with CALD clients, professional status, ethnic status, and language status predict MHP’s awareness of language-related barriers and challenges for themselves.

## Materials and method

### Participants

The sample consisted of 412 mental health professionals who were working in Australia. Most were female (86%), over the age of 30 (75%), psychologists (73%), with full registration (89%), who did not identify as members of an ethnic minority group (83%) and were monolingual (65%). The majority were currently working in a mainstream practice (73%), rather than exclusively with CALD clients, and a large proportion indicated that they had engaged in some form of multicultural training (67%). Table 1 presents detailed participant demographics.

**Table 1.** Descriptive characteristics of participants (*N* = 412).

Variables	*n*	%
Gender		
Male	58	14.1
Female	353	85.7
Other	1	.2
Age		
Between 20–25	33	8.0
Between 26–30	73	17.7
Between 31–40	125	30.3
Between 41–50	83	20.1
Above 50	94	22.8
Ethnic status		
Non-ethnic minority	342	83.0
Ethnic minority	70	17.0
Language status		
Bilingual	142	34.5
Monolingual	270	65.0
Professional Status		
In-training	67	16.3
Fully registered	341	82.8
Missing	4	1.0
Engagement with CALD clients		
Never	16	3.9
Rarely	97	27.4
Occasionally	178	43.2
Often	89	21.6
Always	32	7.8
Multicultural training		
Had training	277	67.2
Did not have training	135	32.8

Participants were recruited using convenience and snowball sampling from public and private mental health services, universities offering postgraduate training for psychologists, counsellors, and social workers, and professional bodies such as the Australian Psychological Society (APS). Despite limitations regarding representativeness, these methods were chosen as the specialist population were not easily identified and can be challenging to recruit (Stratton, 2021). Relevant organisations and individuals were identified by the research team and sent an email invitation containing a link to an online survey hosted on Limesurvey, which took approximately 25 minutes to complete. Eligibility required full or provisional registration as an MHP and prior professional contact with at least one culturally diverse client. Participants could elect to attend a 3-hour workshop on cross-lingual practice or to enter a prize draw for one of two $100 AUD gift vouchers as an incentive. The project received ethical clearance from Griffith University Human ethics committee (PSY/02/15/HREC). Further details are reported here (Garcia de Blakeley et al., 2023).

### Materials

Participants completed demographics including age and gender and the type of practice in which they currently worked (mainstream or specialist CALD service).

#### Language status

Participants indicated whether they were mono or bilingual via a single question “*Do you speak any other language other than English?*” responded to as *Yes* or *No.*

#### Ethnic status

Participants indicated whether they belonged to an ethnic minority via a single question “*Do you identify as a member of an ethnic minority in Australia*” responded to as *Yes* or *No.*

#### Multicultural training

Participants indicated whether they had multicultural training via a single question (*Have you had any training in the area of multicultural mental health or received any training in multicultural competence?”)* responded to as *Yes* or *No.*

#### Engagement with CALD clients

Participants were asked about the frequency of work with CALD clients via a single question “How *often do you work with CALD clients?”* answered on a scale from 1 = *Never* to 5 = *Always*. Those selecting never, rarely or occasionally were coded as having infrequent engagement and those selecting often and always were coded as having frequent engagement.

#### Professional status

Participants indicated their profession from a list of four mental health professions (psychologist, counsellor, social worker, mental health nurse) with an in-training option for each profession (e.g., *Psychologist; In-training psychologist*), and “other”. For the current study, the data was recoded as two groups including “in-training MHPs” and “fully registered MHPs”.

#### Perceptions of Cross-lingual Practice (PCLP)

The PCLP is a 23-item measure consisting of 3-subscales (Garcia de Blakeley et al., 2023): The MHPs’ Perceptions of Self-Competence sub-scale (PSC) consists of 7 items measuring MHPs’ self-assessment of their knowledge and skills and the global assessment of cross-lingual competence (e.g., “*I know how to adjust my language behaviours to cater for clients for whom English is not their native language”)*. The Perceptions of Difficulties faced by Client’s subscale (PDC) consists of 10 items measuring specific language diversity dynamics that could act as barriers during cross-lingual clinical interactions, with implications for psychological assessment and treatment (e.g., *Using the client’s non-native language (English) in therapy hinders clients from accessing certain emotions*). The Perceptions of Barriers for MHPs subscale (PBM) consists of six items measuring MHPs’ awareness of language-related barriers and challenges they are likely to face while conducting psychotherapy with culturally and linguistically diverse clients (e.g., *At times, a client’s strong accent is a barrier to communication*). Each item is rated on a 6-point Likert scale: 1 = Strongly Disagree to 5 = Strongly Agree, and 0 = I do not know. High scores on the PSC sub-scale indicate greater perceived competence while high scores on the PDC and PBM sub-scales greater knowledge and awareness of the barriers and challenges associated with cross-lingual practice. The sub-scales demonstrate good internal reliability (PSC α = .89; PDC α = .88; and PBM α = .76). The PSC sub-scale was weakly positively correlated with the PDC sub-scale but uncorrelated with PBM, while the PDC and PBM were moderately positively correlated (Garcia de Blakeley et al., 2023).

## Results

### Data analytic method

T-tests were used to explore mean differences between the groups on self-perceptions of competence, perceptions of difficulties faced by clients, and perceptions of barriers for MHPs. Hedges’ *g* was used to estimate effect size, Šidák (1967) adjustments were made to account for multiple comparisons, and a Bonferroni correction applied to adjust the alpha for multiple pairwise comparisons on each dependent variable (.05/5 = .01). Hierarchical regression analyses were then conducted to test whether ethnic status, language status, participation in multicultural training, engagement with CALD clients, and professional status predicted self-perceptions of competence, perceptions of difficulties faced by clients, and perceptions of barriers for MHPs after controlling for age and gender. Age and gender were entered as covariates in step 1, and language status, ethnic status, engagement with CALD clients, multicultural training, and professional status entered simultaneously in step 2.

### Exploration of mean level differences between groups

As shown in Table 2, there were significant differences in participant’s self-perceptions of competence whereby MHPs who belonged to an ethnic minority, were bilingual, worked more often with CALD clients, and who had participated in multicultural training perceived their levels of competence to work cross-lingually to be significantly higher than MHPs who did not belong to an ethnic minority, were monolingual, worked with CALD clients infrequently, and who had not participated in multicultural training. There were no significant differences between those who were fully registered and those still in-training (*t* (412) = 2.29, *p* = .023; *g =* 0.31). Similarly, there were no significant differences on any of the variables when comparing MHP’s perceptions of difficulties for clients or barriers for themselves, with the exception that monolingual MHPs (*M =* 21.46, *SD =* 3.38) rated themselves significantly higher than bilingual MHPs (*M* = 20.20, *SD* = 4.20; *t* (412) = 3.09, *p* = .002; *g =* 0.34) on perceptions of barriers for MHPs.

**Table 2.** Mean differences in self-perceptions of competence, perceptions of difficulties faced by clients and perceptions of barriers for MHPs subscale based on demographic, professional, or individual characteristics.

	Variable		
	Ethnic status		
Subscale of the PCLP	Ethnic minority (*n* = 70)	Non-ethnic minority (*n* = 342)	t-statistic, significance, effect size
SPC	*M* = 24.50 (SD = 6.11)	*M* = 20.34 (SD = 5.13)	*t=*5.95, *p* = .001, *g* = 0.84
DPC	*M* = 37.00 (SD = 6.45)	*M* = 36.45 (SD = 5.28)	*t = 0.56, p*=*ns*
PBM	*M* = 20.64 (SD = 4.59)	*M* = 21.10 (SD = 3.53)	*t = 0.79, p*=*ns*
	Language status		
	Bilingual (*n* = 142)	Monolingual (*n* = 270)	
SPC	*M* = 23.88 (SD = 5.26)	*M* = 19.55 (SD = 5.17)	*t=*8.13, *p* = .001, *g* = 0.78
PDC	*M* = 36.46 (SD = 6.52)	*M* = 36.55 (SD = 4.60)	*t = 1.36, p*=*ns*
PBM	*M* = 20.20 (SD = 4.20)	*M* = 21.46 (SD = 3.38)	*t=*3.10, *p* = .002, *g* = 0.12
	Multicultural Training		
	Had training (*n* = 277)	No-training (*n* = 135)	
SPC	*M* = 21.79 (SD = 5.40)	*M* = 19.50 (SD = 5.48)	*t=*4.02, *p* = .001, *g* = 0.42
PDC	*M* = 36.80 (SD = 5.53)	*M* = 35.93 (SD = 4.88)	*t=*1.58, *p*=*ns*
PBM	*M* = 21.13 (SD = 3.55)	*M* = 20.80 (SD = 4.07)	*t=*0.79, *p*=*ns*
	Engagement with CALD clients		
	Frequent (*n* = 121)	Infrequent (*n* = 295)	
SPC	*M* = 23.83 (SD = 5.55)	*M* = 19.89 (SD = 5.10)	*t=*6.96, *p* = .001, *g* = 0.75
PDC	*M* = 37.29 (SD = 6.14)	*M* = 36.20 (SD = 4.94)	*t=*1.90, *p*=*ns*
PBM	*M* = 21.57 (SD = 4.04)	*M* = 20.80 (SD = 3.57)	*t=*1.83, *p*=*ns*
	Professional status		
	Fully registered (*n* = 341)	In-training (*n* = 67)	
SPC	*M* = 21.35 (SD = 5.45)	*M* = 19.67 (SD = 5.67)	*t=*2.29, *p*=*ns*
PDC	*M* = 36.60 (SD = 3.65)	*M* = 35.94 (SD = 4.15)	*t=*0.92, *p*=*ns*
PBM	*M* = 21.12 (SD = 3.65)	*M* = 20.48 (SD = 4.15)	*t=*1.28, *p*=*ns*

*Note*. Self-perceptions of competence (SPC) subscale’s scores ranged from 7 to 35, Perceptions of Difficulties faced by Clients (PDC) subscale’s scores ranged from 10 to 50, and Perceptions of Barriers for MHPs (PBM) ranged from 6 to 30 with higher scores indicating greater competence; CALD = Culturally and Linguistically Diverse; *N* = sample size; PCLP = Perceptions of cross lingual competence; SD = standard deviation.

### Hierarchical multiple regression

Three hierarchical multiple regressions were conducted which tested the predictive utility of each individual characteristic while controlling for the effects of both the covariates and the other predictors in the model. All assumptions for regression were met, and no univariate outliers were identified. Three multivariate outliers were identified but did not exert undue influence and were retained. A summary of each demographic variable’s significant contribution to each dependent variable is presented in Table 3.

**Table 3.** Hierarchical multiple regression for demographic variables predicting self-perceptions of competence, perceptions of difficulties faced by clients and perceptions of barriers for mental health professionals (MHPs) subscale.

	Subscales					
	SPC		PDC		PBM	
Predictor	Δ R^2^	β	Δ R2	β	Δ R2	β
Step 1	.023*		.01		.013	
Age^a^		.130		.010		.075
Gender^b^		.080		.056		.062
Step 2	.312***		.035*		.046**	
Language status		.284***		.050		−.199**
Ethnic status		.039		.010		.023
Professional status		.089*		.049		.055
Engagement with CALD clients		.350***		.164**		.133*
Multicultural training		.161***		.073		.023
Total R^2^	.336***		.044*		.059**	

*Note*. SPC = self-perceptions of competence; PDC = Perceptions of Difficulties faced by Clients; PBM = Perceptions of Barriers for MHPs subscale. ^*a,b*^ *Control* variables; *p < .05, **p.01; ***p < 001.

### Self-perceptions of competence (SPC)

In Step 1, age and gender accounted for 2% of the variance in SPC. Adding language status, ethnic status, multicultural training, professional status, and engagement with CALD clients in Step 2 explained an additional 31% (ΔR^2^ = .31, F(5,395) = 37.14, *p* < .001). In the final model, engagement with CALD clients (β = .35, *p* < .001), language status (β = .28, *p* < .001), multicultural training (β = .16, *p* < .001), and professional status (β = .09, *p* < .05) were significant positive predictors. Overall, the model explained 33% of the variance, F(8,395) = 24.94, *p* < .001.

### Perceptions of difficulties faced by clients (PDC)

In Step 1, age and gender did not significantly predict PDC. Adding language status, ethnic status, professional status, and engagement with CALD clients in Step 2 explained an additional 3.5% of the variance (ΔR^2^ = .035, F(5,395) = 37.14, *p* < .01). In the final model, only engagement with CALD clients (β = .16, *p* < .01) was a significant predictor. Overall, the model accounted for 4% of the variance, F(8,395) = 2.29, *p* < .02.

### Perceptions of barriers for MHPs (PBM)

In Step 1, age and gender did not significantly predict PBM. Adding language status, ethnic status, professional status, and engagement with CALD clients in Step 2 explained an additional 4.6% of the variance (ΔR^2^ = .046, F(5,395) = 3.90, *p* = .01). In the final model, only language status (β = .20, *p* < .01) and engagement with CALD clients (β = .13, *p* < .01) were significant predictors. Overall, the model accounted for 6% of the variance, F(8,395) = 3.12, *p* < .01.

## Discussion

The aim of this study was to explore whether multicultural training, engagement with CALD clients, professional status, ethnic status, and language status were associated with MHPs’ cross-lingual competence. Overall, our findings suggest that there are distinct predictors for self-perceived competence as compared to the more knowledge-based components of competence; difficulties clients might face and barriers caused by language that MHP’s may face.

The first hypothesis was predominantly supported. MHPs who self-identified as belonging to an ethnic minority, were bilingual, worked with CALD clients more frequently, and had engaged in multicultural training perceived themselves as significantly more competent than their counterparts. However, there were no significant differences based on professional status. These findings are consistent with prior research on mental health practitioners’ self-reported multicultural competence (Constantine, 2001; Hill et al., 2013; Sodowsky et al., 1994, 1998) and language-related ability (Costa & Dewaele, 2014; Stevens & Holland, 2008). Similarly, the second hypothesis was only partially supported. Engagement with CALD clients was the strongest predictor of MHPs’ perceived competence, followed by language status, multicultural training, and professional status. Although ethnic minority MHPs reported higher mean levels of perceived competence than their majority counterparts, ethnicity was not a significant predictor when considered alongside other factors. These findings align with Arthur and Januszkowski (2001), who found that frequency of work with CALD clients and multicultural training predicted competence, whereas ethnic minority status did not. Importantly, this suggests that regardless of MHP’s background, engaging in appropriate training and working with diverse clients increases perceived cross-lingual competence.

In terms of the research questions posed regarding the relationship between the individual characteristics and knowledge of difficulties faced by clients and barriers for MHPs, our findings suggested minimal associations. Specifically, results showed that MHPs did not significantly differ in terms of their perceptions of difficulties faced by clients or barriers for MHPs based on their ethnic minority status, professional status, multicultural training, and frequency of engagement with CALD clients. The only exception was that monolingual MHPs reported significantly higher knowledge of barriers for themselves compared to bilingual MHPs. This aligns with literature suggesting monolingual MHPs’ are often aware of the challenges language diversity poses for them, likely due to personal difficulties in cross-lingual work and uncertainty about addressing these barriers without experience in second-language dynamics or adequate training (Costa & Dewaele, 2014; Stevens & Holland, 2008). Such awareness may reflect sensitivity to cross-lingual therapy but also greater discomfort. Importantly, knowing about barriers does not equate to knowing how to overcome them; competence requires strategies developed through insightful practice, training, and supervision. Further research is needed to clarify how factual knowledge of barriers relates to cross-lingual competence and experiences of struggle.

In terms of predictive utility, language status and engagement with CALD clients emerged as predictors of perceptions of barriers for MHPs, but only engagement with CALD clients emerged as predictor of perceptions of difficulties faced by clients. In fact, working with CALD clients was the only individual characteristic in this study that was found to be associated with all three aspects of cross-lingual competence measured by the PCLP’s subscales. Our findings suggest that MHPs who work with CALD clients more often have a higher sense of self-perceived competence and have more factual knowledge of the barriers and challenges of competently engaging cross-lingual work. This is a promising finding, since engagement with CALD clients is a malleable variable that can be systematically increased during post-graduate training, clinical internships and externships, as well as after full registration. However, its contribution to the models was very small (4%−6%), suggesting caution is needed when interpreting findings. It is likely there are other factors beyond those investigated here that may explain MHPs’ differences on levels of factual knowledge. Alternatively, more nuanced measurement of the study variables, such as engagement in multicultural training, may have yielded different results. Further research is needed to investigate what predicts MHPs’ perceptions of challenges/barriers for themselves and clients.

Multicultural training has commonly been recommended to improve cultural competence. However, our findings suggest caution when recommending this as the only strategy for developing competence. Notably, while MHPs who attended multicultural training reported significantly higher self-perceived competence than those who did not, training did not improve their knowledge of client difficulties or barriers they may experience themselves. It is possible that training may foster a sense of competence in addressing cultural and language diversity without providing the specific knowledge needed to manage challenges in cross-lingual practice. Importantly, we did not ask specifically about what was covered in the training MHP’s undertook, nor how much training they had received, which may explain our findings. However, given that most multicultural training does not cover language diversity our findings suggest a need for specific cross-lingual training and the need to further investigate whether training increases an MHP’s actual competence or only perceived competence.

### Practical implications

Our findings challenge the assumptions that the ethnic minority status of an MHP is the most important feature in multicultural and cross-lingual clinical work. Instead, we propose that training specifically focused on cross-lingual competence should be developed and systematically implemented for all MHPs, regardless of their language, ethnic, or professional background.

Our findings also suggest that exposing MHPs to CALD clients more often may increase general levels of cross-lingual competence. To this end, postgraduate training programs should investigate how they can facilitate such exposure while MHPs are still in a supported and supervised environment. However, increased contact must be paired with appropriate training and preparation to avoid unsystematic, trial-and-error engagement with CALD clients, which can heighten practitioners’ uncertainty, stress, and fear of causing cultural harm (Geerlings et al., 2018; Ho & O’Donovan, 2018; Lee & Khawaja, 2013). Such unstructured contact may pose risks to both clients and practitioners, leading to suboptimal assessment, diagnosis, and treatment, and ultimately poor outcomes for the therapeutic dyad (Geerlings et al., 2018).

### Limitations and future directions

The results of this study call for further research to investigate predictors of cross-lingual competence beyond demographic and professional characteristics. Further, many of the constructs were measured using single-item measures that may not capture the nuances of constructs such as ethnic identity, multicultural training, and engagement with CALD clients. A more nuanced examination of these predictors may yield different results and could provide practical guidance to those designing interventions. The sample also predominantly included female psychologists and while representative of the demographic profile of psychologists in Australia (Psychology Board of Australia, 2025b), this along with our sampling strategies may limit generalisability. Finally, the PCLP is a newly developed measure of cross-lingual competence, that despite having excellent psychometric properties (Garcia de Blakeley et al., 2023), has not yet been benchmarked against gold standard measures of competences (i.e., Objective Structured Clinical Examination’s or supervisors’ observations and evaluative reports). Future research is warranted to establish convergent validity and normative data.

## Conclusion

This study is the first to specifically investigate predictors of cross-linguistic competence among Australian MHPs. Our findings demonstrate that engagement with CALD clients had the most consistent positive association with cross-lingual competence, independent of clinicians’ ethnic or language background. Furthermore, multicultural training was associated with practitioners’ self-perceived competence (and likely confidence in working with CALD clients), but caution is needed as training may not equip MHP’s with the nuanced knowledge required to address the specific challenges encountered in cross-lingual practice. Furthermore, monolingual MHPs report greater awareness of barriers for themselves in cross-lingual therapy yet may lack insight into their clients’ perspectives whereas ethnic minority, bilingual and fully registered MHP’s have greater levels of self-perceived competence. These results highlight a potential need for targeted training within professional development frameworks that not only builds confidence but also focuses on knowledge acquisition. In a multicultural context such as Australian there is a strong need to invest in evidence-based strategies that empower all clinicians to deliver equitable and effective care for everyone. Future research should extend and apply these findings by exploring additional predictors, refining assessment tools, and developing professional training to ensure that MHPs are equipped to meet the needs of an increasingly diverse population.

## Data Availability

The participants of this study did not give written consent for their data to be shared publicly, so due to the sensitive nature of the research supporting data is not available.
